# Towards species‐level forecasts of drought‐induced tree mortality risk

**DOI:** 10.1111/nph.18129

**Published:** 2022-04-22

**Authors:** Martin G. De Kauwe, Manon E. B. Sabot, Belinda E. Medlyn, Andrew J. Pitman, Patrick Meir, Lucas A. Cernusak, Rachael V. Gallagher, Anna M. Ukkola, Sami W. Rifai, Brendan Choat

**Affiliations:** ^1^ 1980 School of Biological Sciences University of Bristol Bristol BS8 1TQ UK; ^2^ ARC Centre of Excellence for Climate Extremes Sydney NSW 2052 Australia; ^3^ 7800 Climate Change Research Centre University of New South Wales Sydney NSW 2052 Australia; ^4^ 6489 Hawkesbury Institute for the Environment Western Sydney University Locked Bag 1797 Penrith NSW 2751 Australia; ^5^ School of Geosciences The University of Edinburgh Edinburgh EH9 3FF UK; ^6^ College of Science and Engineering James Cook University Cairns Qld 4878 Australia

**Keywords:** Australia, cavitation resistance, drought tolerance, land surface model, plant hydraulics, species

## Abstract

- Predicting species‐level responses to drought at the landscape scale is critical to reducing uncertainty in future terrestrial carbon and water cycle projections.
- We embedded a stomatal optimisation model in the Community Atmosphere Biosphere Land Exchange (CABLE) land surface model and parameterised the model for 15 canopy dominant eucalypt tree species across South‐Eastern Australia (mean annual precipitation range: 344–1424 mm yr^−1^). We conducted three experiments: applying CABLE to the 2017–2019 drought; a 20% drier drought; and a 20% drier drought with a doubling of atmospheric carbon dioxide (CO_2_).
- The severity of the drought was highlighted as for at least 25% of their distribution ranges, 60% of species experienced leaf water potentials beyond the water potential at which 50% of hydraulic conductivity is lost due to embolism. We identified areas of severe hydraulic stress within‐species’ ranges, but we also pinpointed resilience in species found in predominantly semiarid areas. The importance of the role of CO_2_ in ameliorating drought stress was consistent across species.
- Our results represent an important advance in our capacity to forecast the resilience of individual tree species, providing an evidence base for decision‐making around the resilience of restoration plantings or net‐zero emission strategies.

## Introduction

Droughts, including the co‐occurrence of droughts and heatwaves (Mueller & Seneviratne, 2012; Reichstein *et al*., 2013), have emerged as one of the principal threats to the function of terrestrial ecosystems and are projected to worsen into the future in some regions (Ridder *et al*., 2020; Ukkola *et al*., 2020). These ‘hotter droughts’ – due both to an increase in heatwaves and a background warming of the climate – have been identified as a key driver in reducing plant productivity (Zscheischler *et al*., 2014), growth (Julio Camarero *et al*., 2018; Scharnweber *et al*., 2020) and the terrestrial carbon sink (Yang *et al*., 2018; Green *et al*., 2019). Ultimately, if droughts result in widespread species dieback (Mantgem *et al*., 2009; Peng *et al*., 2011; Mitchell *et al*., 2014), they may lead to sustained changes in biodiversity and community composition (Slik, 2004; Nepstad *et al*., 2007; Anderegg *et al*., 2013), altering land–atmosphere feedbacks (Swann *et al*., 2018) on seasonal to decadal timescales. To develop a predictive framework and better understand the impacts and implications of changes in the timing, severity and spatial extent of climatic extremes, we need to combine insights across scales (McDowell *et al*., 2016), including field ecology (Rowland *et al*., 2015), remote sensing (Bastos *et al*., 2021), modelling and climate science (Williams *et al*., 2020).

Efforts towards understanding the physiological controls of drought‐induced mortality (McDowell *et al*., 2008; Choat *et al*., 2018; Hammond *et al*., 2019) are set against a background of widespread reports of dieback due to drought (Allen *et al*., 2015). Significant progress in our understanding has been enabled by experimental manipulations (Duan *et al*., 2013; Li *et al*., 2018; Venturas *et al*., 2018; Ruehr *et al*., 2019), field‐based surveys and syntheses (Martin‐StPaul *et al*., 2017; Peters *et al*., 2021) and international coordinated networks (e.g. Drought‐Net). More recently, modelling efforts (Bonan *et al*., 2014; Xu *et al*., 2016; Sperry *et al*., 2017; Dewar *et al*., 2018; Kennedy *et al*., 2019; Eller *et al*., 2020; Sabot *et al*., 2020) have emerged that leverage more mechanistic representations of plant hydraulics, allowing us to scale up our understanding and make landscape‐scale predictions (De Kauwe *et al*., 2020). It is worth reflecting however, that models included in the latest Coupled Model Intercomparison Project (CMIP6) and the Global Carbon Budget (Friedlingstein *et al*., 2020) lack these recent model advances, leading to systematic biases in model predictions as water becomes limiting (Ukkola *et al*., 2016a; Trugman *et al*., 2018; Humphrey *et al*., 2021; Teckentrup *et al*., 2021). To project how species responses to drought affect the land carbon sink, land–atmosphere feedbacks and shifts in species’ ranges, we need to incorporate a greater diversity of plant strategies into land surface models (LSMs) and global dynamic vegetation models (De Kauwe *et al*., 2015a). Ultimately, robust predictions of ecosystem resilience to droughts and heatwaves require an understanding of species’ capacities to persist, an understanding of recovery processes/timescales, and the capacity to accommodate interactions with other disturbance agents (i.e. fire; Nolan *et al*., 2020 and/or insects and pathogens; Trowbridge *et al*., 2021).

One emerging challenge is how to better connect hydraulics models to observations, particularly as applications of plant hydraulic formulations are applied at larger scales (regional to global). Two contrasting paradigms exist: bottom‐up and top‐down approaches. Bottom‐up approaches have attempted to use modelling to scale up, either by linking empirical thresholds derived in the field to water deficit simulated by models (Anderegg *et al*., 2015), or by parameterising ‘target’ species (Sperry *et al*., 2019; De Kauwe *et al*., 2020); few have simulated explicit species responses (but please refer to Petit‐Cailleux *et al*., 2021). None of these bottom‐up approaches lend themselves to simple incorporation in LSMs used in CMIP6‐type initiatives, which instead rely on top‐down approaches that group vegetation into plant functional types (PFTs; please refer to Anderegg *et al*. (2022) for a discussion of approaches to capture plant diversity within models). By contrast, top‐down approaches could conceivably be parameterised using satellite data. However recent studies (Konings & Gentine, 2017; Liu *et al*., 2021a) have shown diversity in hydraulics traits (both within and among species) do not readily translate into model PFTs, which may preclude their widespread application in LSMs (Kennedy *et al*., 2019).

Here, our goal was to advance our capacity to assess tree mortality from a species perspective (bottom‐up). De Kauwe *et al*. (2020) previously added a plant hydraulics scheme to Australia’s LSM, Community Atmosphere Biosphere Land Exchange (CABLE) and parameterised the model for five broad vegetation types to identify vulnerability to drought. Extending this approach at the species level is challenging because it requires 10 hydraulic parameters per species (e.g. cuticular conductance, leaf/stem capacitance, sapwood density, etc.), which are hard to obtain outside of experimental conditions. Consequently, in this study, we embed a simplified stomatal optimisation model (Sabot *et al*., 2020) into CABLE that only relies on three hydraulic parameters. We parameterised CABLE for 15 canopy dominant eucalypt tree species (Table 1) originating from a broad precipitation gradient across South‐Eastern Australia (mean annual precipitation range: 344–1424 mm yr^−1^). Between 2017 and 2020, South‐Eastern Australia experienced one of the hottest (Abram *et al*., 2021) and most intense droughts on record (Bureau of Meteorology, 2020), culminating in canopy dieback (Nolan *et al*., 2021) and record‐breaking wildfires (Nolan *et al*., 2020). We used this period to determine which eucalypt species was most vulnerable to hydraulic drought mortality. Finally, we asked how a future more intense drought and an increase in atmospheric carbon dioxide ([CO_2_]) would change predictions of species vulnerability.

**Table 1:** Summary of hydraulic trait parameter values.

Species	*b*(MPa)	*c*(–)	*P*_12_(MPa)	*P*_50_(MPa)	*P*_88_(MPa)	Reference
*E. blakelyi*	5.03	3.36	−2.72	−4.51	−6.12	Li *et al*. (2018)
*E. camaldulensis*	4.10	4.35	−2.56	−3.77	−4.15	Franks *et al*. (1995)
*E. crebra*	5.52	3.08	−2.83	−4.90	−6.41	Bourne *et al*. (2017)
*E. dunnii*	5.56	3.06	−2.84	−4.93	−8.44	Bourne *et al*. (2017)
*E. globulus*	2.55	8.30	−1.99	−2.44	−7.48	Barotto *et al*. (2016)
*E. grandis*	3.58	5.29	−2.43	−3.34	−4.94	Li *et al*. (2018) Bourne *et al*. (2017)
*E. largiflorens*	8.28	3.25	−4.40	−7.40	−9.95	Li *et al*. (2018)
*E. macrorhyncha*	4.40	3.95	−2.61	−4.01	−5.95	Li *et al*. (2018)
*E. melliodora*	5.67	3.02	−2.87	−5.02	−6.65	Li *et al*. (2018)
*E. obliqua*	2.73	7.67	−2.08	−2.60	−3.64	Pritzkow *et al*. (2020)
*E. populnea*	6.41	2.86	−3.12	−5.64	−7.70	Li *et al*. (2018)
*E. saligna*	3.65	5.14	−2.45	−3.40	−5.16	Bourne *et al*. (2017)
*E. sideroxylon*	4.54	3.79	−2.64	−4.12	−5.30	Li *et al*. (2018)
*E. tereticornis*	4.36	3.99	−2.61	−3.98	−5.70	Bourne *et al*. (2017)
*E. viminalis*	3.36	5.80	−2.35	−3.15	−4.43	Li *et al*. (2018)

*b*(MPa) and *c* (unitless) are sensitivity and shape parameters of the plant hydraulic vulnerability curve. *P*_12_, *P*_50_ and *P*_88_ are the water potential inducing a 12%, 50% and 88% loss in hydraulic function, respectively. Note, the value for *Eucalyptus grandis* represents the average trait values taken from the two cited references. [Correction added after first publication 22 April 2022: a reference in the table has been corrected.]

## Materials and Methods

### 2017–2020 Drought

Although the winter and spring of 2016 were wet, dry conditions began across Australia in 2017. Across the Murray–Darling basin, rainfall was substantially below average during 2017–2020 and in some regions of South‐Eastern Australia it was the lowest on record (Supporting Information Fig. S1).

**Fig. 1:**
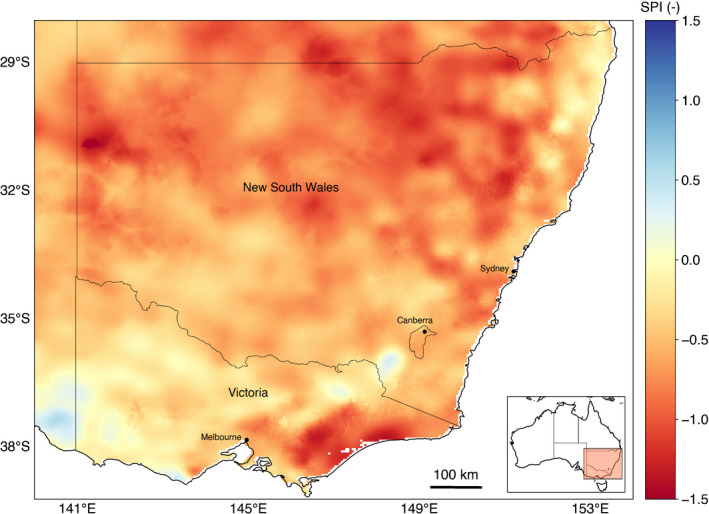
Map of the mean Standardised Precipitation Index (SPI) anomaly during the drought (2017–2019). Anomalies were calculated relative to the historical baseline period of 1911–2016. Note we do not include data from after September 2019 onwards due to the confounding impact of fires across South‐Eastern Australia.

A range of drought definitions exist (Cook *et al*., 2018). Here we only consider the meteorological drought (a deficit of long‐term rainfall) of 2017–2020 in South‐Eastern Australia and the resulting impact on the vegetation (ecological drought), as simulated by the CABLE plant hydraulics scheme (please refer to below). Fig. 1 shows the 6‐month Standardised Precipitation Index (SPI; McKee *et al*., 1993) calculated from the Bureau of Meteorology’s Australian Water Availability Project (AWAP) (Jones *et al*., 2009) precipitation data. A 6‐month SPI represents a ‘medium‐term’ precipitation deficit, often associated with anomalous streamflow and reservoir levels. Fig. 1 shows extensive areas where the SPI exceeded –0.5 and some regions of southern Victoria where it exceeded –1.0. In short, South‐Eastern Australia was subject to widespread and significant meteorological drought during the study period.

### Model description

CABLE is the land surface scheme used in the Australian Community Climate Earth System Simulator (ACCESS, please refer to http://www.accessimulator.org.au; Kowalczyk *et al*., 2006). CABLE can be run offline using prescribed meteorological forcing (Wang *et al*., 2011; De Kauwe *et al*., 2015b; Ukkola *et al*., 2016b; Decker *et al*., 2017; Haverd *et al*., 2018), or fully coupled (Pitman *et al*., 2011; Lorenz *et al*., 2014) within ACCESS. CABLE simulates the surface exchange of carbon, energy and water, representing the vegetation with a single layer, two‐leaf (sunlit/shaded) canopy scheme (Wang & Leuning, 1998), and accounting for within‐canopy turbulence (Raupach, 1994; Raupach *et al*., 1997). The model simulates soil water and heat conduction across six discrete soil layers (ranging to 4.6 m depth), following the Richards equation. The standard model groups the vegetation into 11 PFTs, for global applications. CABLE has the capacity to simulate biogeochemistry (nitrogen and phosphorus) (Wang *et al*., 2010) and vegetation demography model (Haverd *et al*., 2014), but neither was enabled for these simulations as we prescribe the leaf area index (LAI), please refer to the following paragraphs.

The model source code can be accessed freely after registration at https://trac.nci.org.au/trac/cable. In this paper we used CABLE revision 8740.

### Plant hydraulics implementation

Following Sabot *et al*. (2020), we introduced a simplified version of the profit maximisation scheme (Sperry *et al*., 2017) into CABLE to replace the default gas exchange scheme and associated empirical representation of drought stress based on volumetric soil moisture content. Sperry *et al*. (2017) hypothesised that plants regulate their leaf water potential ($\Psi_{\text{leaf}}$; MPa) on an instantaneous basis by weighing a marginal carbon gain (CG) against a hydraulic cost (HC) associated with transpiration, maximising profit as:

(Eqn 1) $${\text{Profit}}_{\max}=\max\left(\text{CG}\left(\Psi_{\text{leaf}}\right)‐\text{HC}\left(\Psi_{\text{leaf}}\right)\right)\in \left[0,1\right]$$

The normalised CG is given by:

(Eqn 2) $$\text{CG}\left(\Psi_{\text{leaf}}\right)=\frac{A(\Psi_{\text{leaf}})}{A_{\max}}\in \left[0,1\right]$$

where *A* (mol m^−2^ s^−1^) is the photosynthetic uptake, expressed as a function of each possible $\Psi_{\text{leaf}}$ between the point of no transpiration (*E*) ($\Psi_{\text{leaf,predawn}}\approx \Psi_{\text{soil}}$, the soil water potential; MPa) and *E* = *E*_crit_, the point of hydraulic failure. *A*_max_ (μmol m^−2^ s^−1^) is the maximum photosynthetic rate over the range of possible $\Psi_{\text{leaf}}$.

To reduce the computational cost of introducing an optimisation scheme into a LSM, we directly link *A*($\Psi_{\text{leaf}}$) to *A* expressed as a function of each possible leaf intercellular CO_2_ concentration (*C*_i_, μmol mol^−1^), *A*(*C*_i_), via the Farquhar model (Farquhar *et al*., 1980) and Fick’s law of diffusion applied to the supply of CO_2_ through stomata. Therefore, instead of co‐optimising *C*_i_ and stomatal conductance for every possible $\Psi_{\text{leaf}}$ (as in Sperry *et al*., 2017), we solve *A* across a sequence (*n* = 1000) of potential *C*_i_, between the CO_2_ compensation point in the absence of mitochondrial respiration ($\Gamma^{*}$, μmol m^−2^ s^−1^) and *C*_s_. Each *A* is associated with a stomatal conductance rate to CO_2_ (*g*_sc_, mol m^−2^ s^−1^) to represent the CO_2_ flux from the leaf surface (*C*_s_, μmol mol^−1^) to the leaf intercellular airspace (*C*_i_, μmol mol^−1^). This in turn, defines the leaf transpiration rate (*E*_leaf_, mmol m^−2^ s^−1^) and then $\Psi_{\text{leaf}}$ (please refer to Eqn 3 below), assuming that the water vapour exchange is proportional to stomatal conductance. This approach trades off a negligible degree of accuracy in the optimal solution as jumps in *C*_i_ were roughly the equivalent of *c*. 0.35 μmol mol^−1^, but significantly improves the computational efficiency which is necessary for LSMs.

The gas exchange calculations are solved iteratively within CABLE’s loop that simulates the leaf temperature that closes the energy balance for the leaf (Wang & Leuning, 1998). Finally, $\Psi_{\text{leaf}}$ is approximated by re‐arranging the steady‐state formulation:

(Eqn 3) $$\Psi_{\text{leaf}}=\Psi_{\text{soil,w}}‐\frac{E_{\text{leaf}}}{k_{\Psi_{\text{leaf}}}}$$

where $\Psi_{\text{soil,w}}$ is the weighted average of the soil water potential (MPa) and $k_{\Psi_{\text{leaf}}}$ is the soil‐to‐canopy conductance (mmol m^−2^ leaf s^−1^ MPa^−1^), that is *k*_max_ evaluated at $\Psi_{\text{leaf}}$.

To reduce parameterisation, we do not solve separate xylem and leaf water potentials (as done in De Kauwe *et al*., 2020). This water potential assumption and our incorporation of the leaf energy balance, are points of distinction from the original Sperry *et al*. (2017) model, with a final difference relating to how we obtain a representative $\Psi_{s}$, please refer to the following paragraph.

For each soil layer, the volumetric water content (*θ*, m^3^ m^−3^) is related to $\Psi_{s}$ following Campbell (1974). To obtain a representative value of the root‐zone $\Psi_{s}$, we weight the average $\Psi_{s}$ for each of the six soil layers by the weighted soil‐to‐root resistance to water uptake of each layer (Williams *et al*., 2001; De Kauwe *et al*., 2015a). This approach weights $\Psi_{s}$ to the upper soil layers when the root‐zone is wet, but shifts towards the deeper soil layers as the soil dries and the soil hydraulic resistance of the layer increases (please refer to De Kauwe *et al*., 2020).

The normalised HC (Eqn 1) is given by:

(Eqn 4) $$\text{HC}\left(\Psi_{\text{leaf}}\right)=\frac{k_{i\max}‐k_{\Psi_{\text{leaf}}}}{k_{i\max}‐k_{\text{crit}}}$$

where $k_{i\max}$(mmol m^−2^ s^−1^ MPa^−1^) is the instantaneous maximum plant hydraulic conductance attenuated by water stress (i.e. *k*_max_ evaluated at $\Psi_{\text{soil}}$), and $k_{\text{crit}}$(mmol m^−2^ s^−1^ MPa^−1^) is the critical hydraulic conductance that characterises hydraulic failure, set to be 5% of $k_{\max}$(Brodribb & Cochard, 2009; Sabot *et al*., 2020).

*k*_*i*max_ and $k_{\Psi_{\text{leaf}}}$are given by the cumulative Weibull distribution (Neufeld *et al*., 1992):

(Eqn 5) $$k\left(\Psi \right)=k_{\max}e^{‐{\left(\frac{\Psi }{b}\right)}^{c}}$$

where Ψ refers to the $\Psi_{s}$ when calculating *k*_*i*max_ and $\Psi_{\text{leaf}}$ for $k_{\Psi_{\text{leaf}}}$. *b* (MPa) and *c* (unitless) are sensitivity and shape parameters of the plant hydraulic vulnerability curve. We assumed that cavitation can be fully recovered following rainfall and recharge of the root‐zone, that is *k* can be fully recovered.

To infer the point of hydraulic failure, we track the percentage loss of hydraulic conductivity (PLC):

(Eqn 6) $$\text{PLC}=100\times \left(1‐\frac{k}{k_{\max}}\right)$$

A strong link has been shown between a threshold corresponding to an 88% loss of xylem hydraulic conductance (*P*_88_) and drought mortality (Barigah *et al*., 2013; Li *et al*., 2015, 2018; Hammond *et al*., 2019). Here, we do not directly equate *P*_88_ with mortality, but instead associate it with the vegetation approaching a point of hydraulic stress consistent with mortality, $\Psi_{\text{thresh}}$. This distinction is important because each grid cell (*c*. 5 km^2^) would contain some trees, not all of which would be dead. To properly associate $\Psi_{\text{thresh}}$ to mortality would require stochastic approaches that are beyond the scope of the study. Please refer to Methods S1; Figs S2–S4 for a description of site validation.

### Model simulations

#### Species distribution

To obtain outputs at the species level we parameterised CABLE for 15 *Eucalyptus* species (please refer to below). Simulations were then run for each *Eucalyptus* species, assuming each species could grow across the domain of South‐Eastern Australia. Model outputs were then filtered (postprocessed) by individual species distribution maps to relate outputs to individual *Eucalyptus* species. Species distribution maps were constructed from spatially referenced species occurrence records accessed from the Atlas of Living Australia for the period 1950–present (Andrew *et al*., 2021). Species occurrence records were first quality controlled to remove erroneous spatial records and to standardise taxon names (Andrew *et al*., 2021). Species distributions were constructed using Poisson point process models (PPMs) using regularised down‐weighted Poisson regression (Renner *et al*., 2015) based on 20 000 background points. PPMs were trained on mean annual temperature (°C), mean diurnal temperature range (°C), annual precipitation (mm), precipitation seasonality (coefficient of variation), annual mean radiation (W m^−2^), aridity index, bedrock depth (m), soil bulk density (fine earth) in kg m^−3^, clay mass fraction (%), silt mass fraction (%) and pH. These climate and soil factors (detailed in Renner *et al*., 2015) were chosen as they reflect major abiotic factors shaping plants growth and nutrition in Australia.

#### Model parameterisation

We used the hydraulic traits (Table 1) of 15 *Eucalyptus* species to parameterise the plant hydraulic vulnerability curve parameters (Eqn. 5). We used two hydraulic traits that describe the xylem pressure that induces a 12%, 50% or 88% loss of hydraulic conductivity due to embolism (i.e. *P*_12_ or *P*_50_, or *P*_88_). Lacking data to parameterise $k_{\max}$by species, we assumed a fixed value of 1.5 mmol m^−2^ s^−1^ MPa^−1^ for each species. This value is in line with estimated values for European species with a mean annual precipitation *c*. 700–1100 mm yr^−1^ (Sabot *et al*., 2020), a range covering 11 of our 15 species. We carried out two experiments to examine the sensitivity to a smaller $k_{\text{max}}$ value (please refer to below). We either used species data to parameterise the maximum carboxylation rate (*V*_cmax_) or used an average across *Eucalyptus* species (Table 2).

**Table 2:** Summary of photosynthetic trait parameter values.

Species	*V*_cmax25_(μmol m^−2^ s^−1^)	Reference
*Eucalyptus blakelyi*	86.88	*
*E. camaldulensis*	111.74	Zhou *et al*. (2016)
*E. crebra*	86.88	*
*E. dunnii*	86.88	*
*E. globulus*	85.55	C. Warren (2004); C. R. Warren (2004)
*E. grandis*	93.71	Leuning *et al*. (1991); Clearwater & Meinzer (2001); Grassi *et al*. (2002)
*E. largiflorens*	86.88	*
*E. macrorhyncha*	86.88	*
*E. melliodora*	86.88	*
*E. bliqua*	86.88	*
*E. populnea*	86.88	*
*E. saligna*	76.67	Ghannoum *et al*. (2010)
*E. sideroxylon*	100.37	Ghannoum *et al*. (2010)
*E. tereticornis*	86.88	*
*E. viminalis*	77.60	C. Warren (2004)

* The average species trait values from cited references in the Table 2 and Cernusak *et al*. (2011); Huang *et al*. (2008) and unpublished data from Drake *et al*. cited in Boer *et al*. (2016).

#### Model forcing

We ran offline simulations for South‐Eastern Australia using gridded, 3‐hourly meteorological forcing of precipitation, downward shortwave and longwave radiation, surface air temperature, surface specific humidity, surface wind speed, surface air pressure and CO_2_. We ran the model over the drought period from January 2017 to August 2019 (spin‐up 2011–2016), at a resolution of 0.05° (*c*. 5 km^2^). We excluded data from September 2019 onwards due to the potential contamination related to large‐scale fires in South‐Eastern Australia. The meteorological data are from the Bureau of Meteorology’s AWAP dataset (Jones *et al*., 2009) and the near‐surface wind data of McVicar *et al*. (2008; McVicar, 2011).

CABLE was run with prescribed LAI based on a climatology (1999–2017) derived from the Copernicus LAI product (http://land.copernicus.eu/global/), regridded from a resolution of 0.01° to 0.05°. By prescribing LAI we avoid the need for a long model spin‐up, only requiring 5 yr to stabilise the soil temperature and root‐zone soil moisture.

Soil properties (e.g. texture, soil hydraulic and thermal characteristics) for CABLE were based on the SoilGrids data (Hengl *et al*., 2017). Data were degraded using local area averaging from 250 m to 0.05° for simulations. As is standard in CABLE, we assumed vertically uniform soil texture based on the weighted average of the 2 m SoilGrids data.

### Experiments

We ran three sets of simulations:

1. A control simulation (‘CTL’), representing the 2017–2019 drought in South‐Eastern Australia.
2. A 20% reduction in the 2017–2019 precipitation to represent a future drier (please refer to below) drought (‘rPPT’).
3. To explore how a CO_2_‐induced change in plant water‐use efficiency (De Kauwe *et al*., 2021) may delay the onset of drought conditions, we combined the rPPT experiment with a doubling of the [CO_2_] to *c*. 800 μmol mol^−1^ (‘eCO_2_ × rPPT’).

Climate model projections of historic and future precipitation contain systematic biases for Australia (Alexander & Arblaster, 2017; Grose *et al*., 2020). Continental mean annual precipitation estimates range from *c*. 200 to 1200 mm yr^−1^, with observed‐derived estimates *c*. 400 mm yr^−1^ (L. Teckentrup *et al*., unpublished). Options to correct precipitation include bias correction and the use of regional climate models to dynamically downscale and generate fine scale climate projections. A preliminary analysis of one corrected dataset (the NSW and ACT Regional Climate Model, NARCliM project) highlighted unrealistic interannual variability in precipitation (e.g. annual precipitation varying between 250 and 2250 mm yr^−1^). Given these marked biases in the use of future climate precipitation forcing, we opted for a simpler, uniform reduction in the AWAP rainfall (i.e. rPPT). Our approach maintains a plausible future experiment but does not account for changes in the distribution of humidity and precipitation deficit. The rPPT experiment also allows us to probe the impact of the original drought, which extended to the end of 2020 but was affected by the 2019–2020 South‐Eastern Australia fires (Nolan *et al*., 2020).

#### Sensitivity experiment

To understand how our model assumptions affected simulated drought risk, we carried out three sensitivity experiments at a single location near Armidale, New South Wales (−30.40°S, 151.60°E) during the 2017–2019 drought. This area was reported as having experienced notable foliage dieback during the drought (Nolan *et al*., 2021). In each experiment, we plotted the change in stomatal conductance (*g*_sw_) as a function of $\Psi_{\text{leaf}}$ and examined the resulting impact on the relative loss of hydraulic conductance. We varied: (1) the critical leaf water potential indicative of maximum xylem hydraulic failure (*k*_crit_); (2) the maximum hydraulic conductance in the soil–plant continuum (*k*_max_); (3) the LAI by increasing it by 40% (eLAI); (4) and the [CO_2_], by doubling it, in combination with a 40% increase in LAI (eCO_2_ × eLAI). To further understand the sensitivity to halving *k*_max_, we also repeated all of the main experiments across South‐Eastern Australia (CTL, rPPT and eCO_2_ × rPPT) with the halved *k*_max_ value.

### Data sets used

#### Satellite data

To evaluate CABLE, we calculated anomaly maps (percent difference) using remote sensing estimates of vegetation optical depth (VOD; 2002–2016 baseline) and the normalised difference vegetation index (NDVI; 2001–2016 baseline). VOD describes the attenuation of microwave wavelengths through vegetation and is most sensitive to above‐ground vegetation water content and changes in leaf/branch biomass (Dijk *et al*., 2013). NDVI quantifies the photosynthetically active radiation that is absorbed by vegetation, and therefore reflects the foliar vegetation state. We used the land parameter data record (LPDR) v.3 VOD product (Du *et al*., 2017), which uses retrievals from the Advanced Microwave Scanning Radiometer for EOS (AMSR‐E) and the Advanced Microwave Scanning Radiometer 2 (AMSR2). For NDVI, we used the MOD13A2 (collection 006) product (https://lpdaac.usgs.gov/products/mod13a2v006/).

### Analysis code

All analysis code is freely available from: https://github.com/mdekauwe/SE_AUS_future_drought_risk_paper.git

## Results

### Minimum water potential

**Fig. 2:**
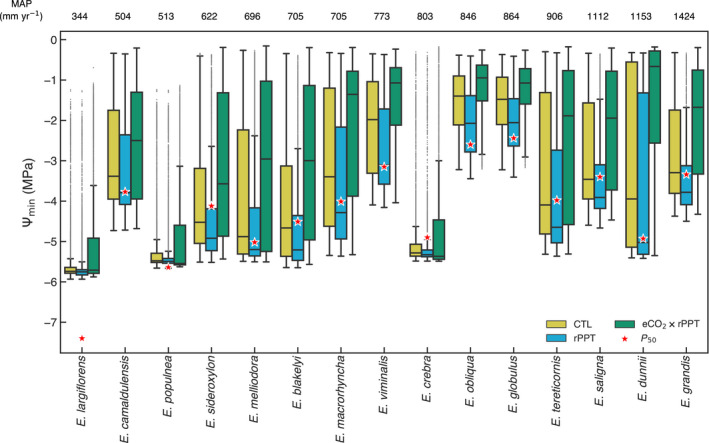
Box and whisker plot (line, median; box, interquartile range) showing the range of simulated minimum leaf water potential ($\Psi_{\min}$) across a species’ distribution for each of the three experiments: 2017–2019 drought (CTL), 20% reduction in rainfall during the drought (rPPT) and 20% reduction in rainfall during the drought in combination with a doubling of atmospheric carbon dioxide, CO_2_ (eCO_2_ × rPPT). The *Eucalyptus* species are ordered from the driest to the wettest, with each species’ mean annual precipitation (MAP) across their range, above each set of box plots. The red stars show each species’ *P*_50_, the xylem pressure inducing 50% loss of hydraulic conductivity due to embolism. Whiskers extend to 1.5 times the interquartile range, with dots outside of the whiskers showing outliers.

**Fig. 3:**
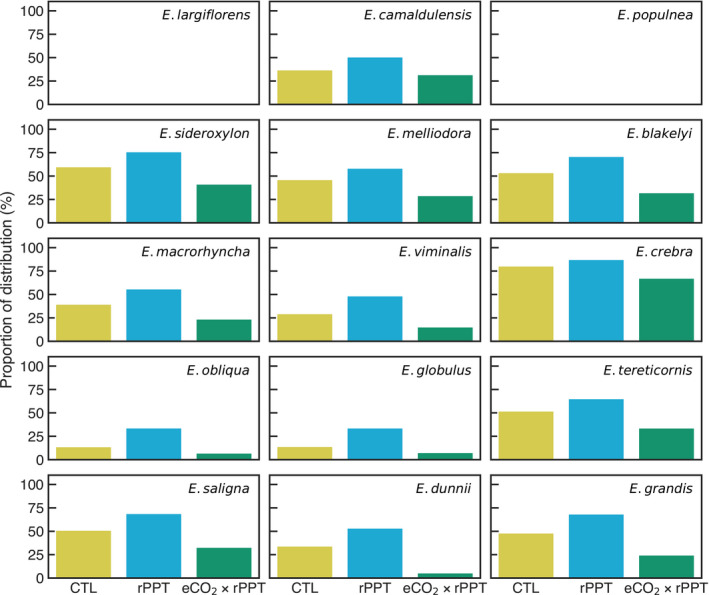
The proportion of each species’ distribution where the simulated mean monthly leaf water potential ($\Psi_{\min}$) dropped below (more negative) than each species’ *P*_50_, the xylem pressure inducing 50% loss of hydraulic conductivity due to embolism. Bars indicate each of the three experiments: 2017–2019 drought (CTL), 20% reduction in rainfall during the drought (rPPT) and 20% reduction in rainfall during the drought in combination with a doubling of atmospheric carbon dioxide, CO_2_ (eCO_2_ × rPPT). The species are ordered from the driest (*Eucalyptus largiflorens*) to the wettest (*E. grandis*), based on mean annual precipitation and matching the order in Fig. 2.

Fig. 2 summarises the impact of the drought expressed as the minimum water potential ($\Psi_{\text{min}}$) reached during the 2017–2019 for each species and for each of our three experiments (Figs S5–S7 show distribution maps). As $\Psi_{\text{min}}$represents the absolute minimum water potential during the drought, it characterises the dehydration tolerance of each species across its distribution. The box and whiskers show the variability of $\Psi_{\text{min}}$ between experiments across a species’ distribution, with the distance between a species’ median and the water potential inducing a 50% loss in hydraulic function (*P*_50_), indicative of the degree of overall stress (please refer to also Fig. 3). The severity of the drought is underlined by several species experiencing levels of stress that pushed the (median of the distribution) $\Psi_{\text{min}}$ close to (*E. populnea*, *E. melliodora*, *E. blakelyi*, *E. tereticornis* and *E. saligna*), or beyond (*E. sideroxylon*, *E. crebra* and *E. grandis*), *P*_50_. The simulated $\Psi_{\text{min}}$ was not strictly related to background dryness (mean annual precipitation), with both mesic and xeric species impacted by the drought (i.e. more negative $\Psi_{\text{min}}$).

Our experiment extending the impact of the drought (rPPT; a proxy for the impact of the real drought that extended to the end of 2020, please refer to the Materials and Methods section) had a greater impact on species with a lower embolism resistance (higher *P*_50_; for example *E. macrorhyncha* and *E. viminalis*) and species with a southern (‘wetter’) distribution (Fig. 2). Overall, the impact of the reduced rainfall was a further reduction in $\Psi_{\text{min}}$relative to CTL (median: −16%, range: −31.7% to −1.5%), although for many species the difference between the CTL and rPPT was limited (e.g. *E. largiflorens*, *E. populnea*, *E. crebra*), implying that some species were already extremely droughted across their distributions. By contrast, doubling the [CO_2_] (rPPT × eCO_2_) had a profound impact on $\Psi_{\text{min}}$, increasing the overall median $\Psi_{\text{min}}$by 32% (range: 7–59%) relative to the rPPT experiment. For most species, doubling [CO_2_] led to a much less negative median $\Psi_{\text{min}}$(e.g. *E. blakelyi*, *E. ideroxylon*, *E. melliodora*, *E. macrorhyncha*), with a few notable exceptions (*E. populnea*, *E. crebra* and *E. largiflorens*).

To emphasise the drought impact on each species, Fig. 3 shows the proportion of each species’ distribution for which the mean monthly $\Psi_{\text{leaf}}$was below *P*_50_. For the CTL experiment, most species (*n* = 9) experienced lower $\Psi_{\text{leaf}}$ than *P*_50_ for at least 25% of their range, with *E. sideroxylon*, *E. blakelyi*, *E. creba* and *E. tereticornis* over 50% of their range. The rPPT experiment had the greatest relative (CTL vs rPPT) impact on *E. obliqua* and *E. globulus*, more than doubling the proportion of the range below *P*_50_. By contrast, doubling [CO_2_] had the least ameliorating impact of drought on *E. crebra* and most on *E. dunnii*.

### Maximum loss of hydraulic conductivity

**Fig. 4:**
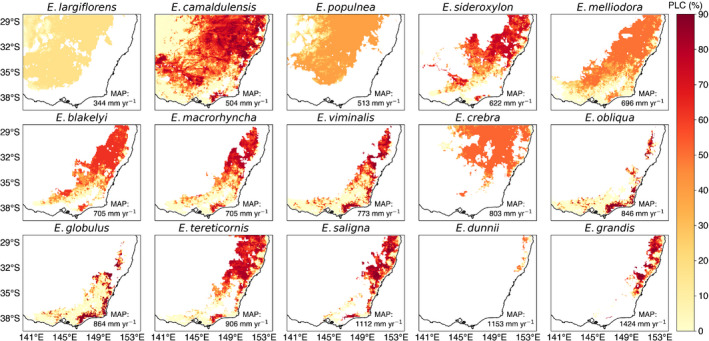
Maps showing the maximum percentage loss of hydraulic conductivity (%) simulated by Community Atmosphere Biosphere Land Exchange (CABLE) during the drought (2017–2019), control (CTL) experiment. The *Eucalyptus* species are ordered from the driest to the wettest, with each species’ mean annual precipitation (MAP) across their range, indicated in each panel. Note we do not include data from after September 2019 due to the confounding impact of fires across South‐Eastern Australia.

Fig. 4 shows the maximum loss of hydraulic conductivity (PLC), for each species across its distribution for the CTL experiment (Figs S8, S9 show the rPPT and eCO_2_ × rPPT maps, respectively). Although the overall loss in conductivity was significant (40%), the impact of drought varied within a species distribution (greatest in the North‐East) and even between species with similar distributions (cf. *E. blakelyi*, *E. macrorhyncha* and *E. viminalis*).

Drought hotspots (PLC = 88%) where drought‐induced hydraulic failure could be anticipated were evident for *E. viminalis*, *E. obliqua*, *E. globulus*, *E. saligna* and *E. grandis*. By contrast, several species appeared resilient to the impacts of the drought (*E. largiflorens*, *E. populnea*, *E melliodora* and *E. crebra*), simulating moderate values of PLC (< 40%). Similarly, several species (*E. melliodora*, *E. crebra*, *E. blakelyi*) had overlapping ranges with hotpots for other species, yet were seemingly resilient due to their more tolerant hydraulic traits. The impact was overall greatest for *E. camaldulensis*, which, despite growing in more arid locations (mean annual precipitation [MAP] across the range = 504 mm yr^−1^), is relatively more sensitive to drought stress than other species (less negative *P*_50_; Fig. 2) in our model simulations. *E. camaldulensis* (‘river red gum’) typically grows along rivers, creeks and waterways and this proximity to a water source is not considered by CABLE, therefore leading to a likely overestimation of the impact of drought.

### Model sensitivities

**Fig. 5:**
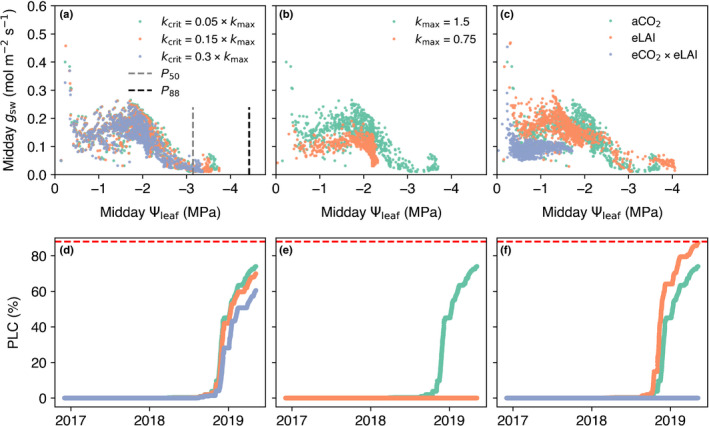
Community Atmosphere Biosphere Land Exchange (CABLE)’s sensitivity to the assumed: (i) critical leaf water potential indicative of maximum xylem hydraulic failure (*k*_crit_; mmol m^−2^ s^−1^ MPa^−1^); (ii) the maximum hydraulic conductance in the soil–plant continuum ($k_{\max}$; mmol m^−2^ s^−1^ MPa^−1^); and a 40% increase in leaf area index (eLAI) and a doubling of the CO_2_ concentration from ambient (aCO_2_) and a 40% increase in leaf area index (eCO_2_ × eLAI). Panels (a–c) show the midday stomatal conductance (*g*_sw_) as a function of midday leaf water potential ($\Psi_{\text{leaf}}$). Panels (d–f) show the corresponding impact of different assumed values in panels (a–c), respectively, on the simulated percentage loss of hydraulic conductance (PLC). The dotted horizontal red line indicates the water potential inducing a critical loss in function (i.e. an 88% loss of hydraulic conductivity). All simulations are from a location near Armidale, New South Wales (30.4°S, 151.6°E).

We identified three key assumptions to which our model results were sensitive. First, given how low some of our simulated species $\Psi_{\text{min}}$values were, we asked whether the optimisation model has sufficient stomatal control as turgor is lost. Examining midday *g*_sw_ as a function of midday $\Psi_{\text{leaf}}$ (Fig. 5a) does suggest that the optimisation scheme does not sufficiently regulate stomata. We can see a few higher (perhaps unrealistic) *g*_sw_ values at more negative midday $\Psi_{\text{leaf}}$ values (< −3 MPa), implying the optimisation scheme obtained marginal ‘profit’ by keeping stomata open, despite strong water‐limiting conditions. Increasing the *k*_crit_ value increases the plant’s stomatal control and therefore decreases the PLC (Fig. 5d).

Second, we did not have data to parameterise *k*_max_, therefore we used the same value for every species. Fig. 5b clearly shows that halving this value significantly reduces evaporative losses and is the difference between CABLE simulating zero PLC and near hydraulic failure (Fig. 5e).

Fig. 2 highlighted the sensitivity $\Psi_{\text{min}}$ values to a doubling of [CO_2_]; however, as LAI was prescribed in our simulation, we have not accounted for the potential effect of [CO_2_]‐induced increases in LAI (Rifai *et al*., 2021). Fig 5c,f shows that a 40% increase in LAI would lead to hydraulic failure (PLC = 88%) relative to the control, as evaporative losses were greater. However, our combined simulation of increased LAI (which ignores the potential for drought‐induced defoliation) and a doubling of [CO_2_] (eCO_2_ × eLAI; Figs 2, S7, S9) suggested that the CO_2_ effect on plant water‐use efficiency cancels out the effect of increased LAI, such that there was no increased risk of hydraulic failure.

**Fig. 6:**
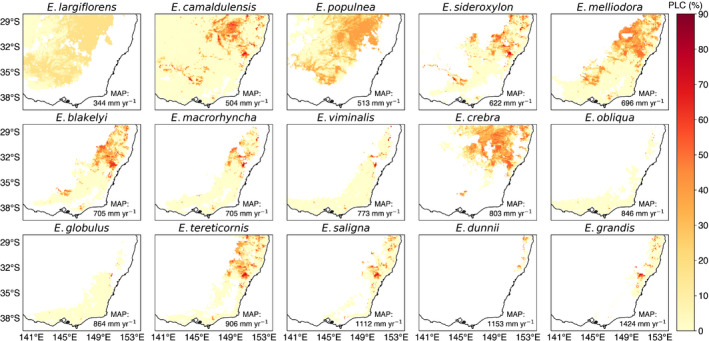
Maps showing the maximum percentage loss of hydraulic conductivity (%) simulated by Community Atmosphere Biosphere Land Exchange (CABLE) when the maximum hydraulic conductance in the soil–plant continuum (*k*_max_) is halved for the 2017–2019 drought (control (CTL) experiment). The *Eucalyptus* species are ordered from the driest to the wettest, with each species’ mean annual precipitation (MAP) across their range, indicated in each panel. Note we do not include data from after September 2019 due to the confounding impact of fires across South‐Eastern Australia.

Focussing further on the sensitivity of *k*_max_, Fig. 6 shows the effect of halving *k*_max_ on the simulated PLC at the landscape scale (Figs S10, S11 show the rPPT and eCO_2_ × rPPT maps, respectively). Comparing Figs 4 and 6, we can see that for all species, the apparent mortality risk is greatly reduced. Despite this, we still see a key hotspot (*c*. 32°S and *c*. 150°E) where PLC exceeds 60% for multiple species (*E. sideroxylon*, *E. blakelyi*, *E. macrorhyncha*, *E. viminalis*, *E. tereticornis*, *E. saligna* and *E. grandis*) and other more widespread reductions in PLC > 30% (*E. populnea*, *E. melliodora* and *E. creba*), despite the reduction in *k*_max_.

### Validation

**Fig. 7:**
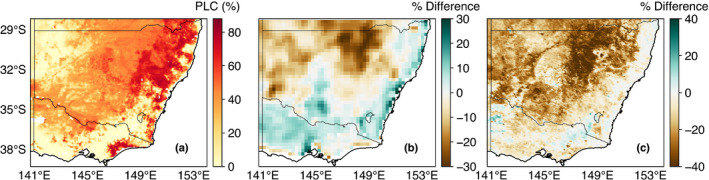
Maps showing (a) the species weighted (by species occurrence in a pixel) maximum loss of hydraulic conductivity simulated by Community Atmosphere Biosphere Land Exchange (CABLE) during the drought (2017–2019). (b) The percentage difference between the mean vegetation optical depth (VOD) during the drought relative to 2002–2016. (c) The percentage difference between the mean normalised difference vegetation index (NDVI) during the drought relative to 2001–2016. Note for all panels, we do not include data from after September 2019 onwards due to the confounding impact of fires across South‐Eastern Australia.

Assessing the accuracy of landscape‐scale, species‐level mortality risk predictions is challenging because: (1) other nontree species (e.g. crops) contribute to the signal; (2) remotely sensed estimates do not directly detect mortality; and (3) knowledge of both species occurrence and species density is required to scale up model simulations. Noting these challenges, we opted to equally weight PLC by species occurrence in a pixel. Fig. 7 therefore shows a crude comparison of PLC simulated by CABLE to remotely sensed anomaly maps of drought impact. CABLE’s simulation of PLC weakly correlated with VOD (*r* = –0.1; Fig. 7a) and NDVI (*r* = –0.21; Fig. 7b). CABLE overstates the extent of the worst‐hit regions (north of 35°S and between 149°E to 151°E), as this hotspot is not evident in the VOD map, and to a lesser extent in the NDVI map. However, there is clear agreement between the region with the greatest anomaly in the satellite maps (north of 32°S and between 147°E to 149°E) and the area where PLC values exceed 50%. CABLE also highlights a hotspot in East Gippsland, in southern Victoria that is present in the SPI map (Fig. 1).

## Discussion

### Identifying how drought risk varies across species ranges

To improve predictions of the impact of future droughts and heatwaves on ecosystems, we need to develop the capacity to accurately forecast near‐term ecological responses (Dietze *et al*., 2018). However, this cannot be achieved with existing modelling frameworks (e.g. CMIP6), as they currently lack key relevant physiological mechanisms and also do not simulate responses at the species level, hindering the potential to inform land management decisions. Our results represent an important advance in the capacity to forecast tree species resilience to drought across South‐Eastern Australia, providing a new evidence base for decision‐making around the long‐term resilience of a broad range of species used in restoration plantings.

CABLE identified several drought hotspots across the ranges of *E. viminalis*, *E. obliqua*, *E. globulus*, *E. saligna* and *E. grandis* (Fig. 4). The rPPT experiment most affected *E. obliqua* and *E. globulus* (Fig. 3), more than doubling the proportion of their range below *P*_50_, whilst for other species, the impact was *c*. 14 percentage points (min = 0; max = 19). The rPPT experiment points to the likely importance of the continued stress imposed by the drought during 2019–2020 bushfires. Our simulations suggested considerable hydraulic failure risk for *E. camaldulensis*, although whether this risk emerged in reality relates to subsurface water availability and extraction in rivers and creeks that our model does not capture. CABLE highlighted resilience to drought in species that are found predominantly in semiarid areas such as *E. largiflorens* and *E. populnea*. CABLE pinpointed the role of hydraulic traits in conferring resilience for species with overlapping distributions (cf. *E. blakelyi* to *E. saligna*) subject to marked precipitation deficits.

Although we did identify uncertainty to our assumed *k*_max_ value (Figs 5, 6), plant hydraulic conductances (per unit leaf area) (De Kauwe *et al*., 2020) for Australian species are consistent with our assumed value (even for arid species), implying our sensitivity experiment is an uncommon scenario. Furthermore, the improved simulations of carbon and water fluxes during periods of water stress at Australian flux sites (Figs S2–S4) and the qualitative spatial agreement between our weighted simulations and satellite derived VOD (cf. Figs 4, 5) provide confidence in the robustness of our model simulations to our assumed *k*_max_.

Notably, our results differ from an application of CABLE with a different plant hydraulics scheme (De Kauwe *et al*., 2020). First De Kauwe *et al*. (2020) identified greater risk of hydraulic failure in the most arid regions (e.g. north of 32°S and west of 145°E) when compared with this study. In both studies, the vegetation type (‘semiarid woodland’) and species covering most of that region (*E. largiflorens*) had resilient hydraulic traits (*P*_50_ < –7 MPa). One point of difference was the higher assumed plant hydraulic conductance (based on experimental data), which may have exacerbated the depletion of soil water in the earlier study. A second difference relates to the role of *g*_min_ (cuticular conductance) that was used in the ‘second’ drought phase in that version of the model (Choat *et al*., 2018). This implies that in the De Kauwe *et al*. (2020) study, complete stomatal closure was simulated due to high vapour pressure deficit (and a continued loss of water via *g*_min_ after stomatal closure, please refer to the following paragraphs), which was not the case with the profit maximisation approach in this study. Capturing the correct stomatal sensitivity as vapour pressure deficit increases is emerging as a key knowledge gap across models, particularly at high vapour pressure deficit (Yang *et al*., 2019). Sabot *et al*. (2022) demonstrated wide variability in the sensitivity of stomatal conductance to vapour pressure deficit across the latest generation of optimal stomatal models (Wolf *et al*., 2016; Sperry *et al*., 2017; Dewar *et al*., 2018; Eller *et al*., 2018). However, as the sensitivity to vapour pressure deficit emerges from the parameterisation of the hydraulic vulnerability curve and the optimisation target, this uncertainty cannot be easily constrained with observations.

### Stomatal control

We hypothesised that the negative drop in $\Psi_{\text{min}}$ (i.e. close to or below the *P*_50_; Fig. 2) may relate to degree of stomatal control in the profit maximisation model (Fig. 5a,d). In earlier work, Sabot *et al*. (2020) identified the importance of the *k*_crit_ parameter for determining the overall stomatal control of the profit maximisation model. This parameter is uncertain, varying by species in relation to the point of complete hydraulic failure, which makes it hard to determine *a priori*. Our results showed potentially unrealistic stomatal control at very negative $\Psi_{\text{leaf}}$ values, where the model increased *g*_sw_ to achieve marginal CGs (Fig. 6a). By contrast, for *Eucalyptus* species, observations point to a strong relationship between the xylem water potential at 90% stomatal closure and the xylem water potential at the inception of xylem cavitation (*P*_12_) (Li *et al*., 2018), implying strong stomatal control. Whilst increasing *k*_crit_ would mute this behaviour, it would not completely stop it, so this remains an area for further analysis.

Alternatively, one could hypothesise that the apparent lack of stomatal control reflects the fact that we are not also attenuating *V*_cmax_ through ‘non‐stomatal limitations’ (Zhou *et al*., 2013) as soil water becomes limiting. In our simulations, a greater direct constraint on photosynthesis would reduce the marginal benefit delivered by opening stomata at very negative $\Psi_{\text{ leaf}}$ values. Previous studies using empirical, rather than optimisation models, have demonstrated the need to capture both stomatal and non‐stomatal limitations during drought (Keenan *et al*., 2010; Zhou *et al*., 2013; De Kauwe *et al*., 2015a).

Similarly, eucalypt access to groundwater (Christina *et al*., 2017; Zolfaghar *et al*., 2017) reflects an important water source that may reduce simulated vulnerability during droughts. Recently, Mu *et al*. (2021) showed that incorporating a groundwater scheme into CABLE (without plant hydraulics) increased transpiration by *c*. 100 mm yr^−1^ during the 2017–2019 drought, predominantly by reducing vertical drainage. Future model developments that link improvements in plant hydraulics to improvements in subsurface hydrology, including the role of deeper root water access (via tap roots, but please refer to Pivovaroff *et al*., 2021), are needed.

An outstanding question for the new generation of stomatal optimisation models (Wolf *et al*., 2016; Sperry *et al*., 2017; Dewar *et al*., 2018; Eller *et al*., 2018) is whether they are capturing the correct degree of stomatal control, or instead if this reflects other process gaps (e.g. non‐stomatal limitations, groundwater, etc.). Specifically, one could ask whether an apparent lack of sufficient control should be expected given these models are linking fast processes (i.e. stomatal opening/closure) to slow processes (i.e. investment in architecture to avoid hydraulic failure), via the cost function (Wolf *et al*., 2016; Sperry *et al*., 2017). More data is needed to determine whether avoiding embolism (represented via the vulnerability curve) is the primary control on stomatal closure, or whether other primary, active controls need to be represented (e.g. abscisic acid accumulation Farquhar & Sharkey, 1982). To emphasise this point, Martin‐StPaul *et al*. (2017) reported a very small range in the point of stomatal closure relative to a range of *P*_50_ values across species, implying additional stomatal regulation may be required.

### The role of [CO_2_] in ameliorating plant drought stress

Reduced stomatal conductance in response to rising [CO_2_] and therefore, increasing soil water content (‘water savings’) has long been hypothesised as a mechanism by which plants may ameliorate the impact of drought (Medlyn *et al*., 2001); however, the evidence is equivocal (De Kauwe *et al*., 2021). For all but three of our species (*E. largiflorens*, *E. populnea* and *E. crebra*), doubling [CO_2_] offset (via increased water‐use efficiency) a considerable proportion of the negative effect of a further reduction in rainfall across South‐Eastern Australia (rPPT). The apparent lack of sensitivity of these three species (*E. largiflorens*, *E. populnea* and *E. crebra*) in fact reflects the duration of moisture stress across their distribution. That is, where the duration of water stress conditions was prolonged (and severe), the capacity of increased plant water‐use efficiency to ameliorate drought stress was negligible. This result can be seen most clearly either by contrasting distribution medians in Fig. 2, or by comparing the lack of change in PLC between the rPPT (Fig. S8) and eCO_2_ × rPPT (Fig. S9) experiments. By contrast, for the other species, it is striking that the increase in $\Psi_{\text{min}}$ was typically found in the southern (typically wetter) parts of distribution ranges (cf. Figs S6, S7), implying a specific role of CO_2_ in ameliorating stress.

Our results are consistent with modelling approaches that have considered responses under future climate. Sperry *et al*. (2019) used a more complicated implementation of the profit maximisation approach applied across continental USA and found a strong role for elevated [CO_2_] in offsetting drought effects. They also showed that future temperature rises could negate these benefits, which we did not test in our future simulations (although our CTL simulation did include high temperatures associated with the drought, particularly in January 2019). Cochard *et al*. (2021) simulated lethal embolism rates for a single oak tree species under a future Representative Concentration Pathways scenario (very high emissions, RCP8.5), attributing the driver to increasing vapour pressure deficit (please refer to below). This strong link to vapour pressure deficit is likely to reflect the assumed temperature sensitivity of cuticular conductance in their model, although support for a link between cuticular conductance and temperature is an important issue to resolve (please refer to Slot *et al*., 2021). Anderegg *et al*. (2015) derived an empirical threshold between observed mortality in *P. tremuloides* and climatic water deficit to determine the likely future timescale of mortality based on coupled climate models. However, this type of approach does not allow for plant responses to [CO_2_] to affect the time point at which this threshold is reached, which may overstate risk. Future work is still required to determine whether models are capturing the correct sensitivity to [CO_2_] when projecting the role of drought stress. This remains one area for which it is particularly challenging to scale from manipulation experiments to models, due to the numerous real‐world interactions (please refer to De Kauwe *et al*., 2021).

### How to parameterise *k*_max_

One important simplification we made in our simulations was to assume that species had the same *k*_max_: this is unrealistic given the breadth of arid conditions occupied across the species distributions. Accurate parameterisation of this key parameter at the ecosystem scale is important, but unfortunately this is not a trait that is routinely reported. Theoretically, where sap flow or eddy covariance data capture individual species evaporative fluxes, the maximum conductance of a plant could be determined. Unfortunately, few eddy covariance sites cover the domain of interest in this study and/or have species that overlap with those for which hydraulic traits have been characterised (Wombat State Forest being the exception – *E. obliqua*).

Alternatively, Sperry *et al*. (2017) hypothesised that the optimal *k*_max_ should be associated with *V*_cmax_, which is an attractive solution as a relationship between maximum water conductance and maximum CG makes theoretical sense. Nevertheless, it remains unclear exactly how *k*_max_ could be directly obtained from *V*_cmax_ data. Sabot *et al*. (2020) tested the hypothesis that *k*_max_ should reflect long‐term site climate (both average and extreme conditions). Whilst their results tended to support this link to long‐term climate, high variability in derived *k*_max_ estimates at moderate MAP ranges (i.e. 700–800 mm yr^−1^) implies that further research is still needed. In all likelihood diversity in *k*_max_ simply reflects a further axis by which hydraulic strategies trade off between and among species. Nevertheless, deriving *k*_max_ as Sabot *et al*. (2020) did, is likely to reflect a more realistic route forwards than simply assuming fixed values, as we did in this study. A further option might be to leverage recent efforts to link VOD to vegetation water content (Liu *et al*., 2021b), utilising this decadal timeseries to determine *k*_max_. Unfortunately microwave‐derived estimates of VOD are extremely coarse (please refer to Fig. 7) and their interpretation is hindered by changes in leaf wetness (Xu *et al*., 2021) and canopy biomass (Momen *et al*., 2017). This may make it hard to relate directly to individual species but may work adequately for coarser vegetation simulations.

### Role of cuticular conductance, *g*_min_

Choat *et al*. (2018) argued that evaporative loss via cuticular conductance was an important control point in the pathway of plants towards hydraulic failure. Although an earlier study with CABLE incorporated *g*_min_ in the hydraulics implementation (De Kauwe *et al*., 2020), we did not use this here for two reasons. First, it represents a further parameter to determine for each species. The values used by De Kauwe *et al*. (2020) ranged from 0.25 to 0.8 mmol m^−2^ s^−1^, implying that fixing the value across species was not straightforward. As noted above, the impact of *g*_min_ across the more arid regions in the earlier study (De Kauwe *et al*., 2020) was significant and may have been overstated (too high a *g*_min_). Further data to inform model parameterisation of *g*_min_ are much needed and may be aided by recent advances (Márquez *et al*., 2022). Second, as our simulations have demonstrated, the profit maximisation already simulates very negative $\Psi_{\text{min}}$ values in extreme drought. Consequently, there is likely to be limited sensitivity to the role of *g*_min_ in this model compared with a stomatal model that more strongly attenuates evaporation as root‐zone water becomes limiting. Future work that revisits the degree of stomatal control in optimal stomatal models is perhaps needed first before connections are made to *g*_min_. Nevertheless, given the physiological importance of *g*_min_ to predicting drought mortality, this is a key future avenue of research.

### Future directions

In this study, we explored resilience to drought by using a bottom‐up approach to integrate traits and climate to gain insight into species vulnerability across their distribution. However, drought susceptibility also varies within a species (Tuomela, 1997; Taeger *et al*., 2013), which is a key axis of variation that we are unable to currently capture with modelling, severely limiting our predictive capacity as the climate changes. Although several compilations of physiological and hydraulic traits exist (Choat *et al*., 2012; Martin‐StPaul *et al*., 2017; Liu *et al*., 2019; Falster *et al*., 2021; Peters *et al*., 2021), few adequately sample variations within species across the environments they occupy (Rowland *et al*., 2021; Trugman *et al*., 2021). In the absence of direct observations of interspecific trait variation, modelling approaches that explore trait sensitivity (De Kauwe *et al*., 2020; Papastefanou *et al*., 2020) offer one possibility to bridge this gap. Nevertheless, these approaches are unlikely to capture important strategy trade‐offs and their link to climate of origin. Alternatively, Konings & Gentine (2017) demonstrated that spatial variations in isohydricity could be derived from microwave estimates of vegetation water content. Conceivably these data may offer a pathway to better capture inter‐and intraspecific variation in drought responses at the ecosystem scale, directly relevant to global models (please refer to also Konings *et al*. (2021) for a review). Nevertheless, microwave estimates are coarse (> 25 km), hampering comparison with field‐based hydraulic traits; therefore, if used to parameterise models, we could run the risk of conflating within‐species vs among‐species drought risk.

## Author contributions

MGDK designed the research in consultation with BEM, AJP, PM and LAC. MGDK and MEBS developed and implemented the model. RVG generated species distribution maps. AU calculated the precipitation drought metrics. SWR processed the satellite data. MGDK and MEBS collected species traits with input from BC. MGDK carried out all the analysis and wrote the original manuscript draft. All authors contributed to reviewing and editing the final manuscript.

## Supporting information


**Fig. S1** South‐Eastern Australia’s rainfall deciles for January 2017 to August 2019.
**Fig. S2** A comparison between fluxes simulated by CABLE with (hydraulics) and without (Control) the plant hydraulics module for gross primary productivity (GPP) and latent heat flux (LE) at the Tumbarumba fluxnet site during a pronounced period of water stress.
**Fig. S3** A comparison between fluxes simulated by CABLE with (hydraulics) and without (Control) the plant hydraulics module for gross primary productivity (GPP) and latent heat flux (LE) at the Wombat State Forest fluxnet site during a pronounced period of water stress.
**Fig. S4** A comparison between fluxes simulated by CABLE with (hydraulics) and without (Control) the plant hydraulics module for gross primary productivity (GPP) and latent heat flux (LE) at the Whroo fluxnet site during a pronounced period of water stress.
**Fig. S5** Maps showing the minimum leaf water potential ($\Psi_{\text{min}}$) simulated by CABLE during the drought (CTL: 2017–2019).
**Fig. S6** Maps showing the minimum leaf water potential ($\Psi_{\text{min}}$) simulated by CABLE during the drought with a 20% reduction in rainfall (rPPT: 2017–2019).
**Fig. S7** Maps showing the minimum leaf water potential ($\Psi_{\text{min}}$) simulated by CABLE during the drought with a 20% reduction in rainfall and a doubling of the atmospheric carbon dioxide concentration (eCO_2_ × rPPT: 2017–2019).
**Fig. S8** Maps showing the maximum percentage loss of hydraulic conductivity (%) simulated by CABLE during the drought (2017–2019), rPPT experiment.
**Fig. S9** Maps showing the maximum percentage loss of hydraulic conductivity (%) simulated by CABLE during the drought (2017–2019), eCO_2_ × rPPT experiment.
**Fig. S10** Maps showing the relative maximum percentage loss of hydraulic conductivity (%) simulated by CABLE when the maximum hydraulic conductance in the soil–plant continuum (*k*_max_) is halved for the 2017–2019 drought (rPPT experiment).
**Fig. S11** Maps showing the relative maximum percentage loss of hydraulic conductivity (%) simulated by CABLE when the maximum hydraulic conductance in the soil–plant continuum (*k*_max_) is halved for the 2017–2019 drought (eCO_2_ × rPPT experiment).
**Methods S1** Site validation of CABLE at Ozflux sites.
Please note: Wiley Blackwell are not responsible for the content or functionality of any Supporting Information supplied by the authors. Any queries (other than missing material) should be directed to the *New Phytologist* Central Office.

## Data Availability

The model source code can be accessed freely after registration at https://trac.nci.org.au/trac/cable. In this paper we used CABLE revision 8740. All analysis code is freely available from: https://github.com/mdekauwe/SE_AUS_future_drought_risk_paper.git.
