# Supplementary material for: Towards species‐level forecasts of drought‐induced tree mortality risk
Source: New Phytol. 2022 Apr 22;235(1):94–110. doi: 10.1111/nph.18129 (PMC9321630; doi:10.1111/nph.18129)
Supplement: Supplementary file 1 — Fig. S1 South‐Eastern Australia’s rainfall deciles for January 2017 to August 2019. Fig. S2 A comparison between fluxes simulated by CABLE with (hydraulics) and without (Control) the plant hydraulics module for gross primary productivity (GPP) and latent heat flux (LE) at the Tumbarumba fluxnet site during a pronounced period of water stress. Fig. S3 A comparison between fluxes simulated by CABLE with (hydraulics) and without (Control) the plant hydraulics module for gross primary productivity (GPP) and latent heat flux (LE) at the Wombat State Forest fluxnet site during a pronounced period of water stress. Fig. S4 A comparison between fluxes simulated by CABLE with (hydraulics) and without (Control) the plant hydraulics module for gross primary productivity (GPP) and latent heat flux (LE) at the Whroo fluxnet site during a pronounced period of water stress. Fig. S5 Maps showing the minimum leaf water potential (Ψmin) simulated by CABLE during the drought (CTL: 2017–2019). Fig. S6 Maps showing the minimum leaf water potential (Ψmin) simulated by CABLE during the drought with a 20% reduction in rainfall (rPPT: 2017–2019). Fig. S7 Maps showing the minimum leaf water potential (Ψmin) simulated by CABLE during the drought with a 20% reduction in rainfall and a doubling of the atmospheric carbon dioxide concentration (eCO2 × rPPT: 2017–2019). Fig. S8 Maps showing the maximum percentage loss of hydraulic conductivity (%) simulated by CABLE during the drought (2017–2019), rPPT experiment. Fig. S9 Maps showing the maximum percentage loss of hydraulic conductivity (%) simulated by CABLE during the drought (2017–2019), eCO2 × rPPT experiment. Fig. S10 Maps showing the relative maximum percentage loss of hydraulic conductivity (%) simulated by CABLE when the maximum hydraulic conductance in the soil–plant continuum (k max) is halved for the 2017–2019 drought (rPPT experiment). Fig. S11 Maps showing the relative maximum percentage loss of hydraulic conductivity (%) simulated by [file NPH-235-94-s001.pdf]

New Phytologist

New Phytologist Supporting Information

Article title: Towards species-level forecasts of drought-induced tree mortality risk
Authors: Martin G. De Kauwe, Manon E. B. Sabot, Belinda E. Medlyn, Andrew J. Pitman, Patrick Meir, Lucas A. Cernusak, Rachael V. Gallagher, Anna M. Ukkola, Sami W. Rifai and Brendan Choat
Article acceptance date: 28 March 2022

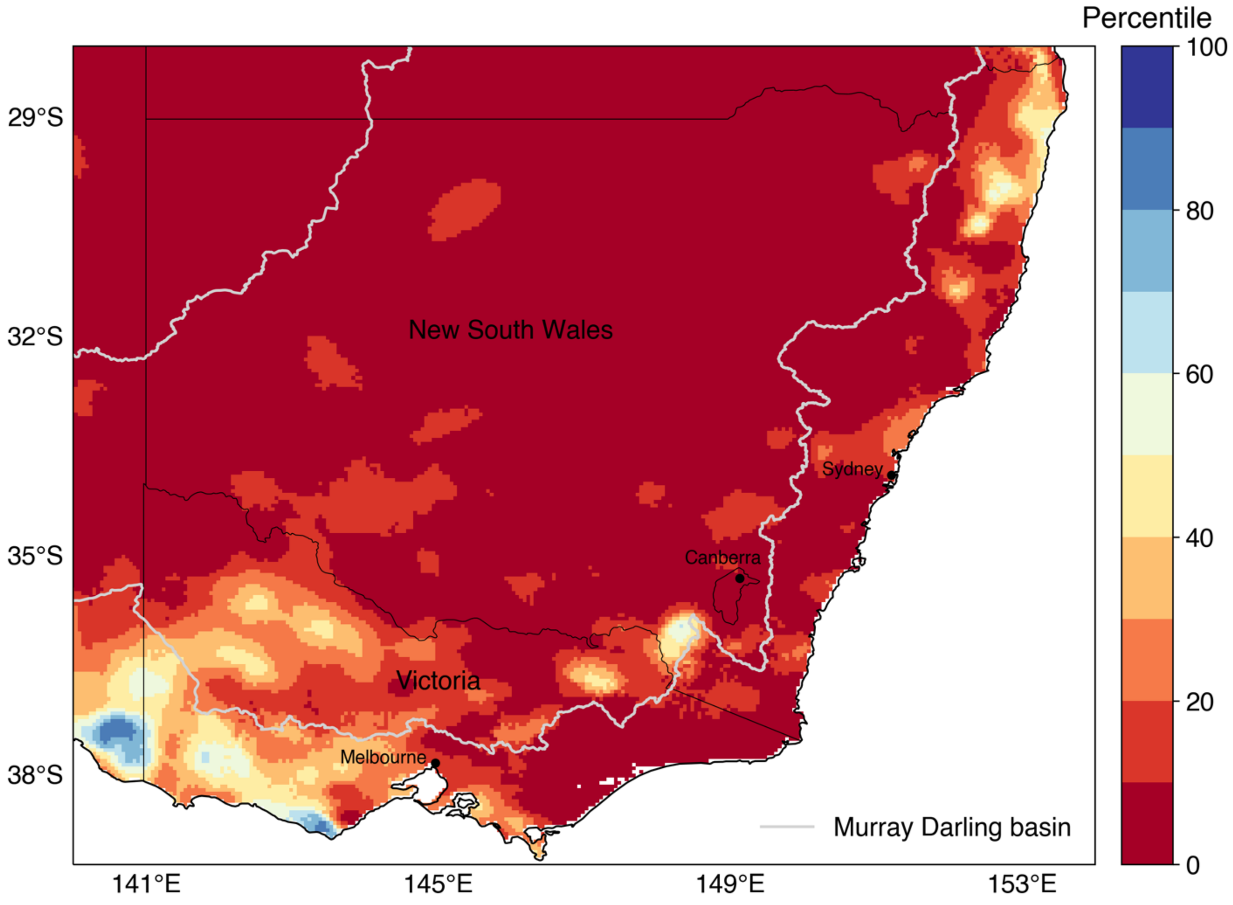

Fig. S1 South East Australia's rainfall deciles for January 2017 to August 2019. The map shows the average rainfall during 2017–2019 ranked against historic records (1901–2016) expressed as a percentile.

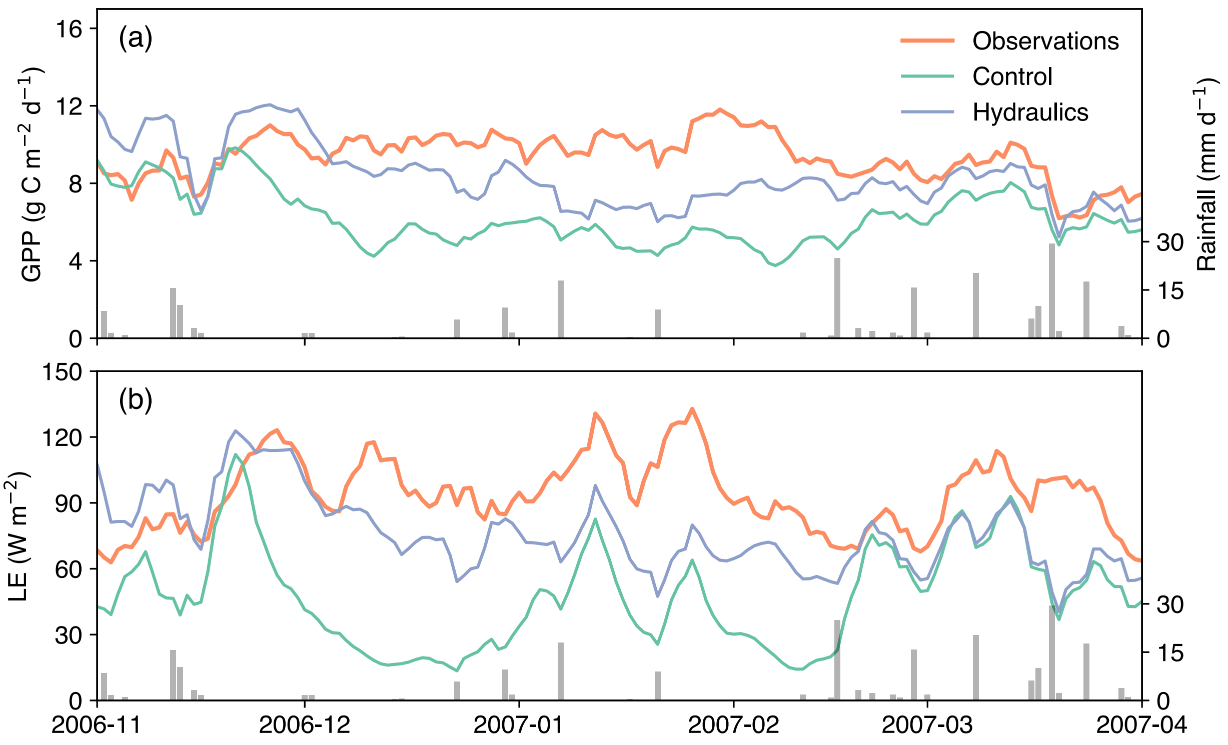

Fig. S2 A comparison between fluxes simulated by CABLE with (hydraulics) and without (Control) the plant hydraulics module for (a) gross primary productivity (GPP) and (b) latent heat flux (LE) at the Tumbarumba fluxnet site during a pronounced period of water stress. The data have been smoothed with a 5-day moving window to aid visualisation.

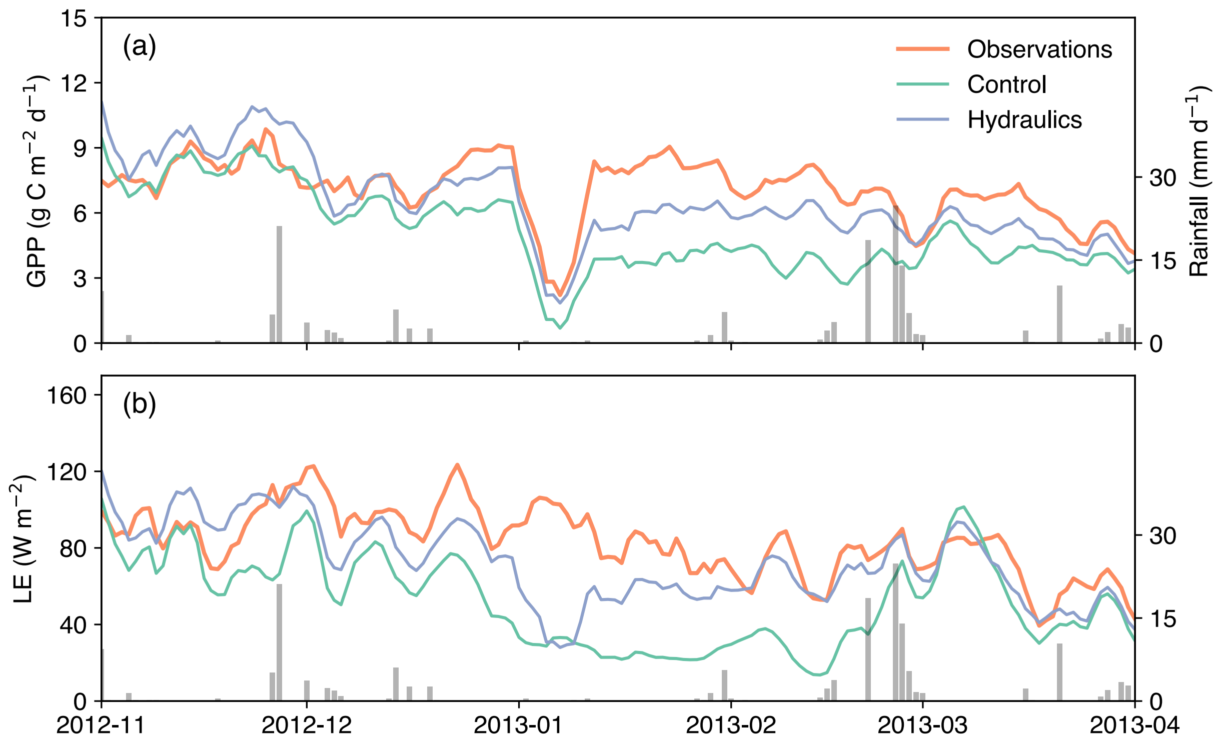

Fig. S3 A comparison between fluxes simulated by CABLE with (hydraulics) and without (Control) the plant hydraulics module for (a) gross primary productivity (GPP) and (b) latent heat flux (LE) at the Wombat State Forest fluxnet site during a pronounced period of water stress. The data have been smoothed with a 5-day moving window to aid visualisation.

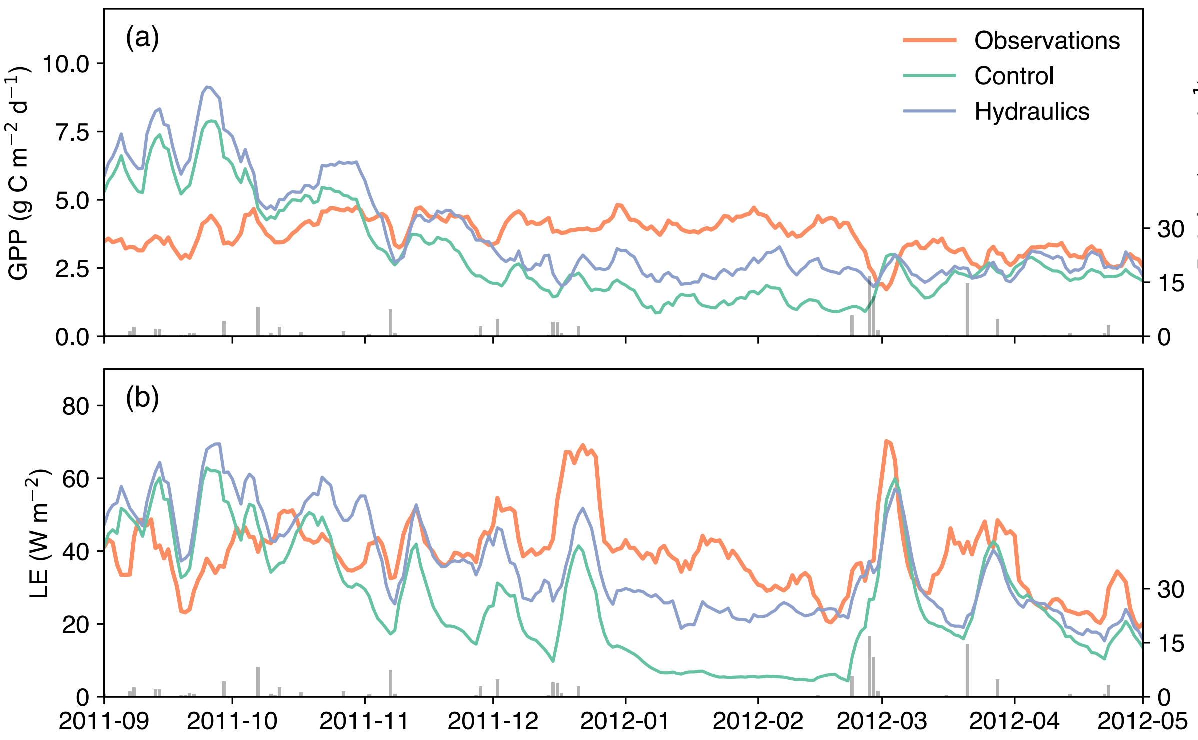

Fig. S4 A comparison between fluxes simulated by CABLE with (hydraulics) and without (Control) the plant hydraulics module for (a) gross primary productivity (GPP) and (b) latent heat flux (LE) at the Whroo fluxnet site during a pronounced period of water stress. The data have been smoothed with a 5-day moving window to aid visualisation.

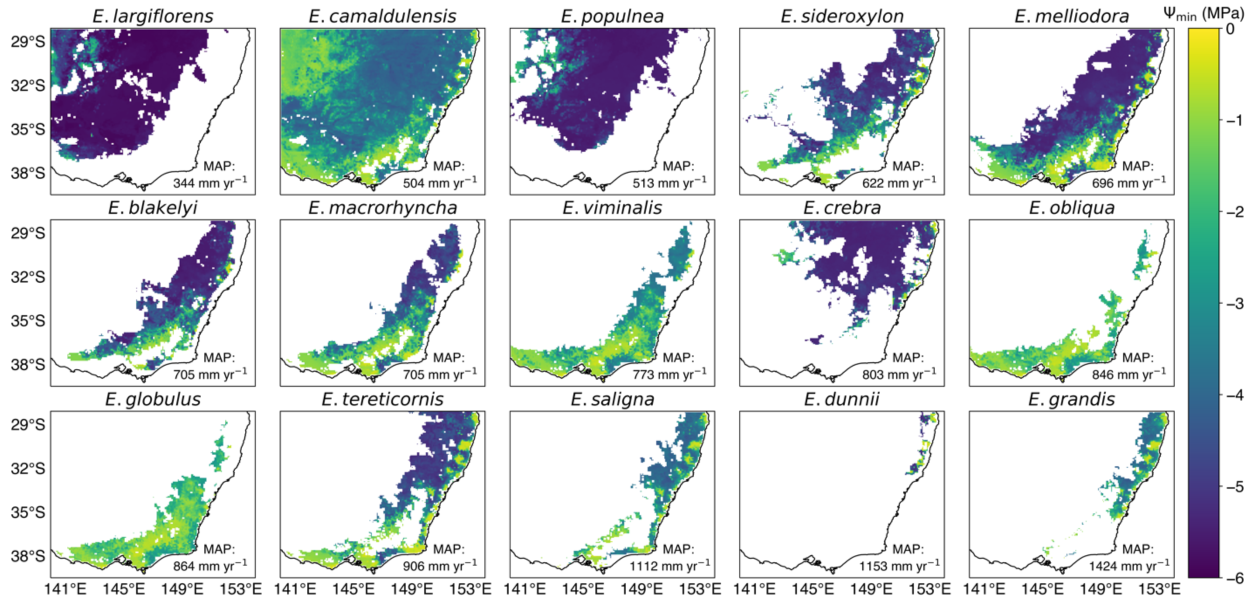

Fig. S5 Maps showing the minimum leaf water potential (Ψ_{min} simulated by CABLE during the drought (CTL: 2017–2019). The species are ordered from the driest to the wettest, with each species' mean annual precipitation (MAP) across their range, indicated in each panel. Note we do not include data from after September 2019 due to the confounding impact of fires across South East Australia.

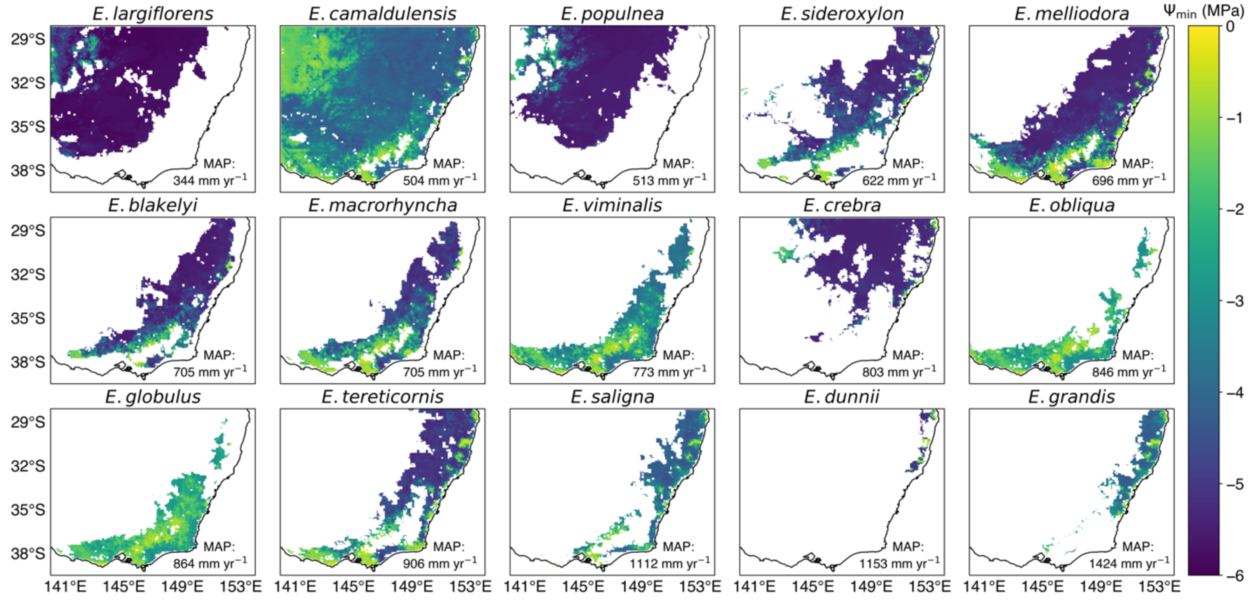

Fig. S6 Maps showing the minimum leaf water potential (Ψ_{min} simulated by CABLE during the drought with a 20% reduction in rainfall (rPPT: 2017–2019). The species are ordered from the driest to the wettest, with each species' mean annual precipitation (MAP) across their range, indicated in each panel. Note we do not include data from after September 2019 due to the confounding impact of fires across South East Australia.

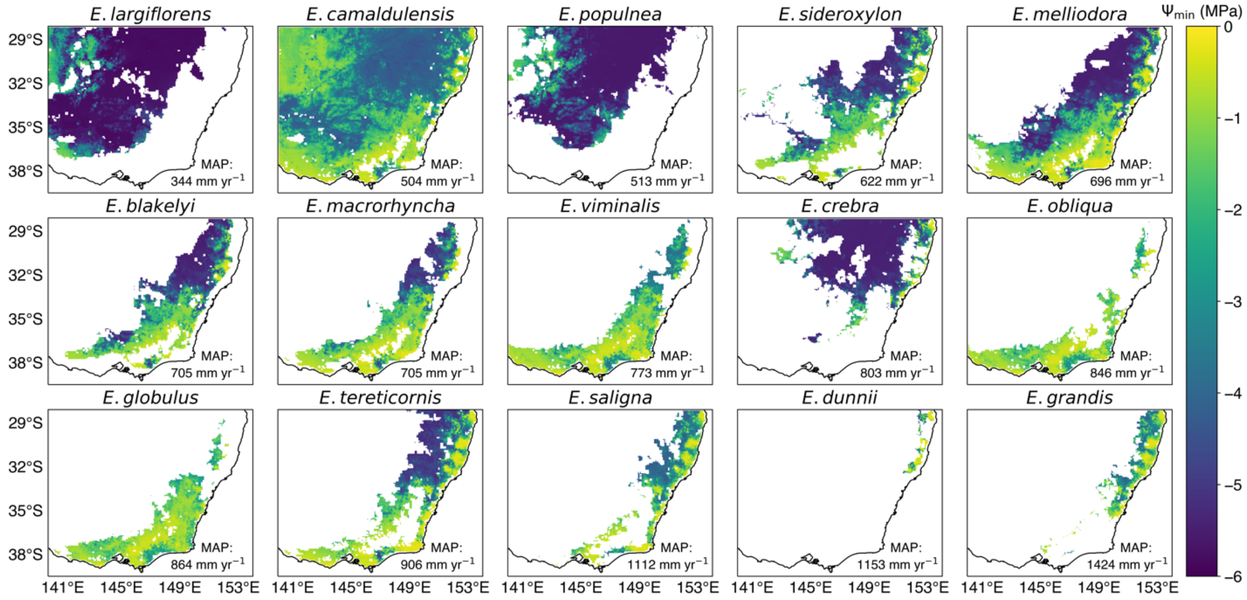

Fig. S7 Maps showing the minimum leaf water potential (Ψ_{min} simulated by CABLE during the drought with a 20% reduction in rainfall and a doubling of the atmospheric carbon dioxide concentration ($eCO_2 \times$ rPPT: 2017–2019). The species are ordered from the driest to the wettest, with each species' mean annual precipitation (MAP) across their range, indicated in each panel. Note we do not include data from after September 2019 due to the confounding impact of fires across South East Australia.

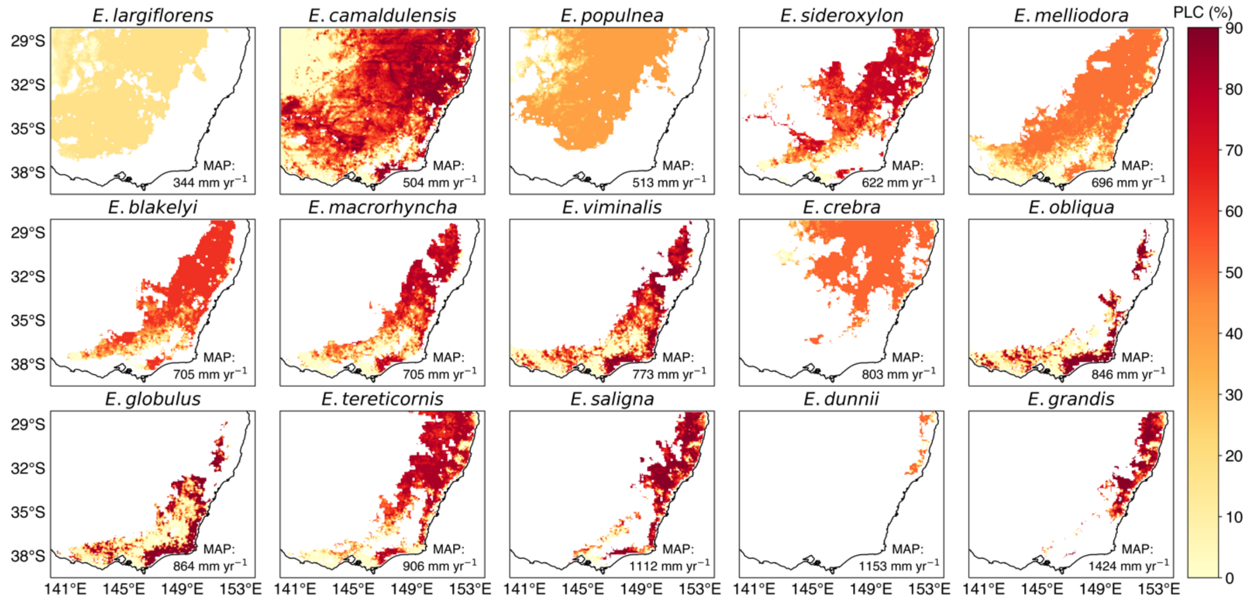

Fig. S8 Maps showing the maximum percentage loss of hydraulic conductivity (%) simulated by CABLE during the drought (2017–2019), rPPT experiment. The species are ordered from the driest to the wettest, with each species' mean annual precipitation (MAP) across their range, indicated in each panel. Note we do not include data from after September 2019 due to the confounding impact of fires across South East Australia.

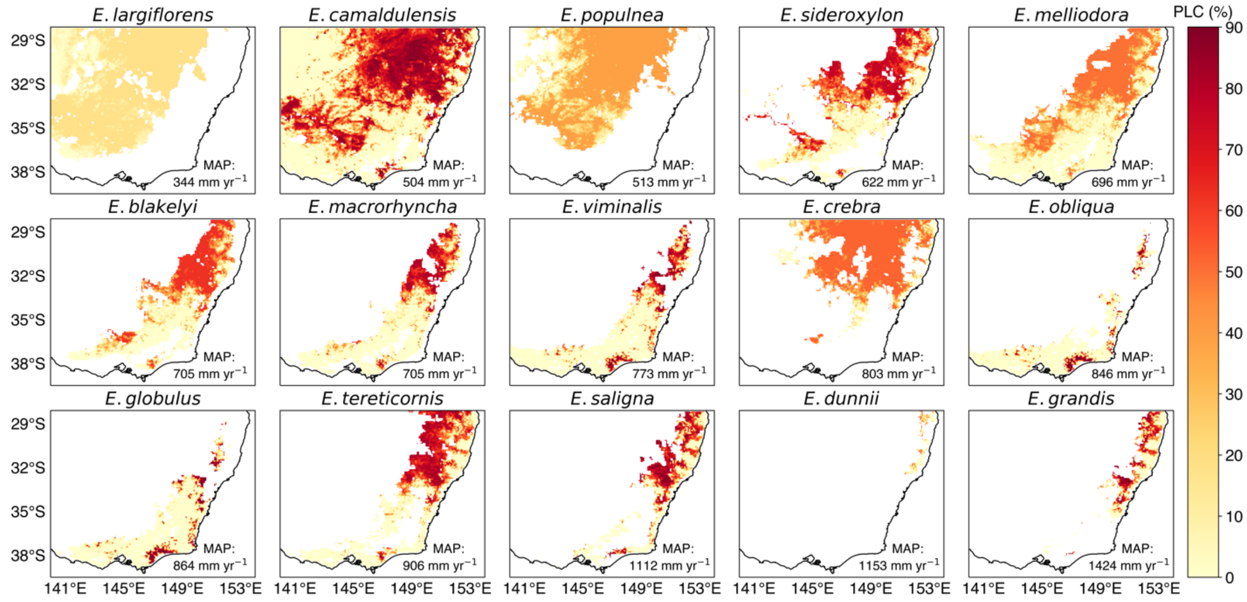

Fig. S9 Maps showing the maximum percentage loss of hydraulic conductivity (%) simulated by CABLE during the drought (2017–2019), $eCO_2 \times$ rPPT experiment. The species are ordered from the driest to the wettest, with each species' mean annual precipitation (MAP) across their range, indicated in each panel. Note we do not include data from after September 2019 due to the confounding impact of fires across South East Australia.

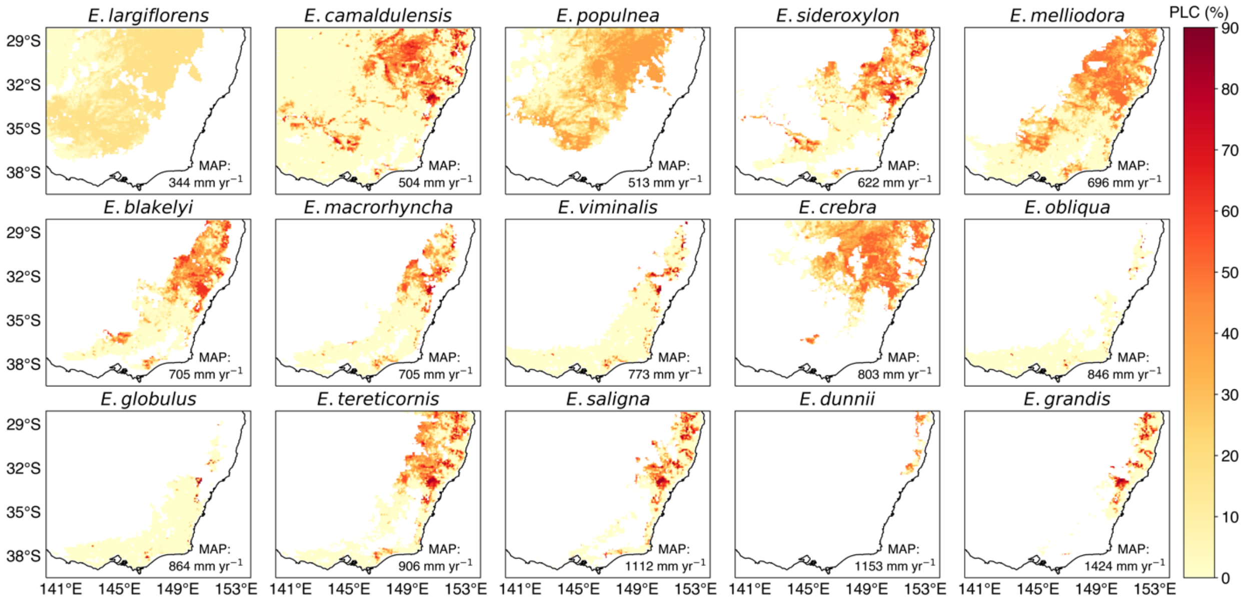

Fig. S10 Maps showing the relative maximum percentage loss of hydraulic conductivity (%) simulated by CABLE when the maximum hydraulic conductance in the soil–plant continuum (k_{max}) is halved for the 2017–2019 drought (rPPT experiment). The species are ordered from the driest to the wettest, with each species' mean annual precipitation (MAP) across their range, indicated in each panel. Note we do not include data from after September 2019 due to the confounding impact of fires across South-East Australia.

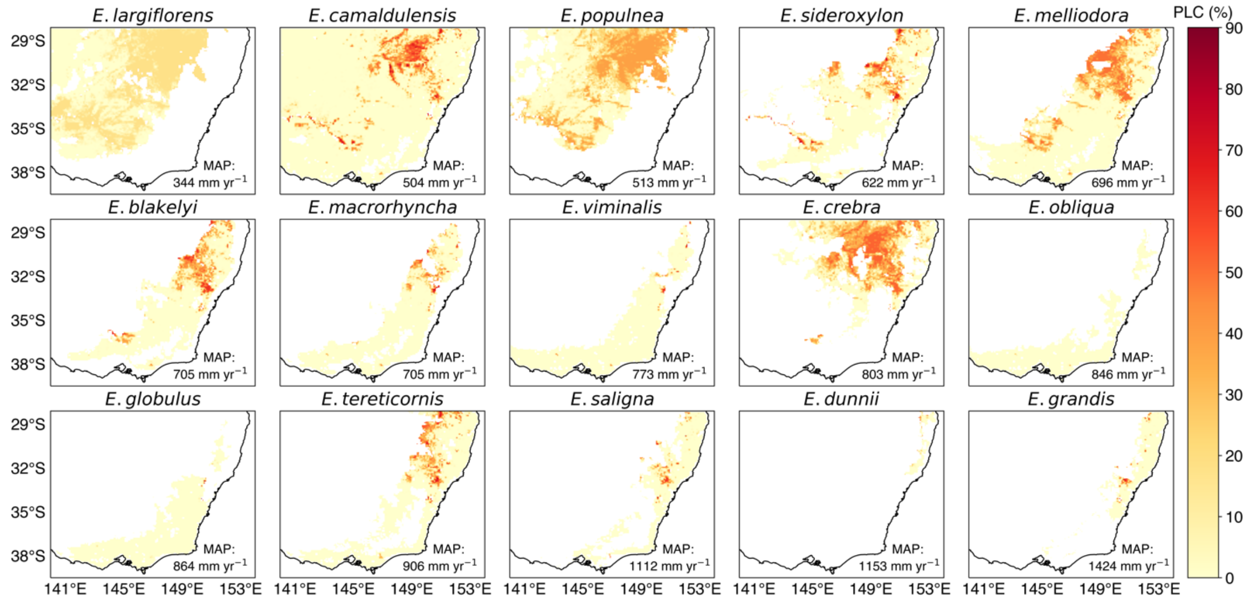

Fig. S11 Maps showing the relative maximum percentage loss of hydraulic conductivity (%) simulated by CABLE when the maximum hydraulic conductance in the soil–plant continuum (k_{max}) is halved for the 2017–2019 drought (eCO_2 × rPPT experiment). The species are ordered from the driest to the wettest, with each species' mean annual precipitation (MAP) across their range, indicated in each panel. Note we do not include data from after September 2019 due to the confounding impact of fires across South-East Australia.

New Phytologist

Methods S1 Site validation of CABLE at Ozflux sites

The integration of plant hydraulics approaches in CABLE has been evaluated in a series of studies (De Kauwe *et al.*, 2015b; Sabot *et al.*, 2020; De Kauwe *et al.*, 2020). Figure S2-S4 shows the evaluation of the plant hydraulics implementation in CABLE at three Australian woodland eddy covariance sites: Tumbarumba (35.65°S, 148.15°E; Keith *et al.* (2012)), the Wombat state forest (37.42°S, 144.09°E; Griebel *et al.* (2016)) and Whroo (36.67°S, 145.03°E; McHugh *et al.* (2017)). We used Level 6 gap-filled OzFlux (http://www.ozflux.org.au) data following Isaac *et al.* (2017). Flux data were pre-processed using the FluxnetLSM R package Ukkola *et al.* (2017) and screened to retain measured and good-quality gap-filled data. As we did not have site specific traits, we paramaterised the model using the *E. obliqua* traits, as this species distribution range overlapped all three sites. We note that *E. obliqua* is one of the dominant species found at Wombat State Forest (along with *E. radiata*, *E. rubida)*). At all sites, the plant hydraulics scheme improves both the simulated carbon and water fluxes (relative to the default CABLE model), with remaining biases likely related to sub-surface hydrology (see Mu *et al.* (2021a) for a comprehensive assessment of biases in CABLE's sub-surface hydrology).

References:

De Kauwe MG, Kala J, Lin Y-S, Pitman AJ, Medlyn BE, Duursma RA, Abramowitz G, Wang Y-P, Miralles DG. **2015a**. A test of an optimal stomatal conductance scheme within the CABLE land surface model. *Geoscientific Model Development* **8**: 431–452.

De Kauwe MG, Medlyn BE, Ukkola AM, Mu M, Sabot MEB, Pitman AJ, Meir P, Cernusak LA, Rifai SW, Choat B, *et al.* 2020. Identifying areas at risk of drought-induced tree mortality across South-Eastern Australia. *Global Change Biology* **26**: 5716–5733.

De Kauwe MG, Zhou S-X, Medlyn BE, Pitman AJ, Wang Y-P, Duursma RA, Prentice IC. **2015b**. Do land surface models need to include differential plant species responses to drought? Examining model predictions across a mesic-xeric gradient in Europe. *Biogeosciences* **12**: 7503–7518.

Griebel A, Bennett LT, Metzen D, Cleverly J, Burba G, Arndt SK. **2016**. Effects of inhomogeneities within the flux footprint on the interpretation of seasonal, annual, and interannual ecosystem carbon exchange. *Agricultural and Forest Meteorology* **221**: 50–60.

Isaac P, Cleverly J, McHugh I, van Gorsel E, Ewenz C, Beringer J. **2017**. OzFlux data: network integration from collection to curation. *Biogeosciences* **14**: 2903–2928.

Keith H, van Gorsel E, Jacobsen KL, Cleugh HA. **2012**. Dynamics of carbon exchange in a Eucalyptus forest in response to interacting disturbance factors. *Agricultural and Forest Meteorology* **153**: 67–81.

McHugh ID, Beringer J, Cunningham SC, Baker PJ, Cavagnaro TR, Mac Nally R, Thompson RM. **2017**. Interactions between nocturnal turbulent flux, storage and advection at an "ideal" eucalypt woodland site. *Biogeosciences* **14**: 3027–3050.

Mu M, De Kauwe MG, Ukkola AM, Pitman AJ, Gimeno TE, Medlyn BE, Or D, Yang J, Ellsworth DS. **2021**. Evaluating a land surface model at a water-limited site: implications for land surface contributions to droughts and heatwaves. *Hydrology and Earth System Sciences* **25**: 447–471.

Sabot MEB, De Kauwe MG, Pitman AJ, Medlyn BE, Verhoef A, Ukkola AM, Abramowitz G. **2020**. Plant profit maximization improves predictions of European forest responses to drought. *New Phytologist* **226**: 1638–1655.

Ukkola AM, Haughton N, De Kauwe MG, Abramowitz G, Pitman AJ. **2017**. FluxnetLSM R package (v1.0): a community tool for processing FLUXNET data for use in land surface modelling. *Geoscientific Model Development* **10**: 3379–3390.
